# Strength Is in Numbers: Can Concordant Artificial Listeners Improve Prediction of Emotion from Speech?

**DOI:** 10.1371/journal.pone.0161752

**Published:** 2016-08-26

**Authors:** Eugenio Martinelli, Arianna Mencattini, Elena Daprati, Corrado Di Natale

**Affiliations:** 1 Department of Electronic Engineering, University of Rome Tor Vergata, Rome, Italy; 2 Department of System Medicine and CBMS, University of Rome Tor Vergata, Rome, Italy; Max Planck Institute for Human Cognitive and Brain Sciences, GERMANY

## Abstract

Humans can communicate their emotions by modulating facial expressions or the tone of their voice. Albeit numerous applications exist that enable machines to read facial emotions and recognize the content of verbal messages, methods for speech emotion recognition are still in their infancy. Yet, fast and reliable applications for emotion recognition are the obvious advancement of present ‘intelligent personal assistants’, and may have countless applications in diagnostics, rehabilitation and research. Taking inspiration from the dynamics of human group decision-making, we devised a novel speech emotion recognition system that applies, for the first time, a semi-supervised prediction model based on consensus. Three tests were carried out to compare this algorithm with traditional approaches. Labeling performances relative to a public database of spontaneous speeches are reported. The novel system appears to be fast, robust and less computationally demanding than traditional methods, allowing for easier implementation in portable voice-analyzers (as used in rehabilitation, research, industry, etc.) and for applications in the research domain (such as real-time pairing of stimuli to participants’ emotional state, selective/differential data collection based on emotional content, etc.).

## Introduction

One of the most irritating features of virtual receptionists is their being utterly impermeable to the emotional outbursts of callers, who, consequently, feel more neglected and less satisfied than when interacting with human attendants. Indeed, despite complexity of the non-verbal signals conveyed by the voice, humans easily recognize them, and react accordingly. Conversely, machines do not detect the emotional information embedded in the voice and, consequently, the human partner may become annoyed by the apparent lack of empathy. Thus, it is not surprising that speech emotion recognition systems (SER) have recently become of interest to the domain of human-machine interfaces [1–2], although their application is relevant also for treatment of psychiatric and neurologic conditions affecting the emotional sphere (e.g. autism [3–4] Parkinson Disease [5–7], mood disorders [8]).

Regardless the application, a priority for SER systems is obtaining fast, online labeling of long speech sequences [9]. On this respect, a promising opportunity comes from the domain of machine learning and, specifically, from semi-supervised and unsupervised learning machines, which are typically used when a huge amount of data requires labeling (i.e. in diagnostic imaging, remote sensing imaging, etc.)[10–11]. In standard supervised learning methods, an initial model is firstly trained using a set of (pre-)labeled data. This model is then employed to automatically describe unlabeled data, using the machine-generated examples to improve prediction capabilities. In contrast, in semi-supervised learning approaches, an initial model provides estimates for some of the unlabeled data. Then, these machine-labeled examples are added to the training set, the model is retrained, and the process iterated. In both cases the learning phase is usually performed on descriptors extracted from long speech sequences coming from different speakers [12–13]. Consequently, accuracy and speed of the system is largely dependent on the amount of computational and data resources involved: the larger the amount the longer (and more cumbersome) the computation. As a result, most of the available SER systems show limited performances, precluding their inclusion in applications where they could be extraordinarily useful [14–16], such as smartphones and tablets.

In human behavior, strength is often in numbers. Truth of the “*vox populi*” concept was first demonstrated by Galton in the early 20^th^ century [17]. At an annual fair, about eight hundred visitors estimated the weight of an ox. Individual guesses ranged widely and often wrongly. In contrast, the middlemost estimate of the distribution, i.e. the *vox populi*, was correct to within 1 per cent of the real value. That large groups are better problem-solvers than single individuals is now well established in a number of domains (see for instance [18]). This “*wisdom of the crowds*” mainly comes from the large diversity of opinions and has proved an efficient feature in many areas, including that of diagnostics, improving fracture classification reliability [19]. In a similar way, we reasoned that by simulating a ‘*wise crowd*’ we could devise an efficient model of group decision-making that could be applied to a novel SER system.

**Fig 1:**
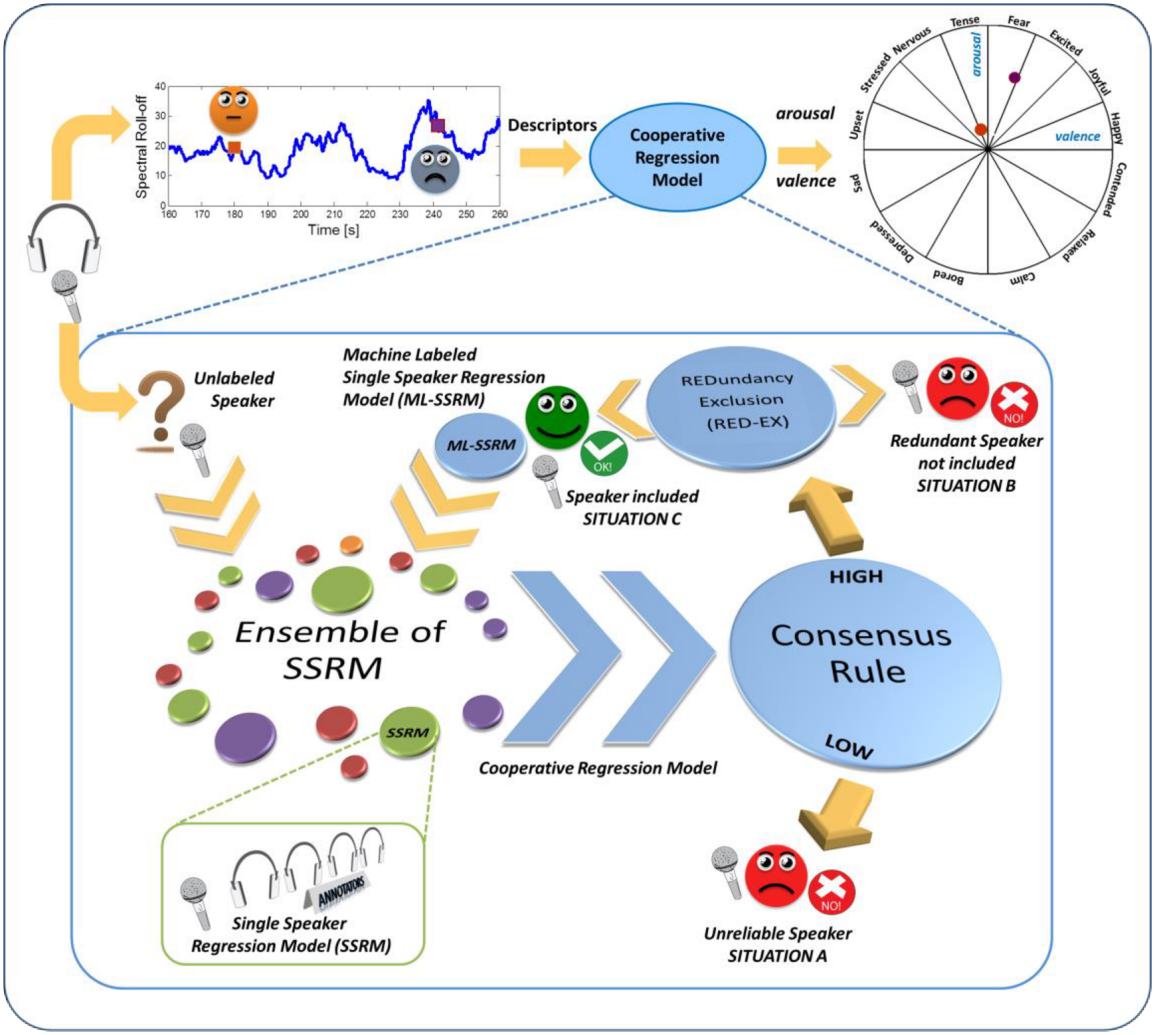
Schematic description of the prediction system. *Upper part*: speech is continuously recorded in a naturalistic environment. In small time windows, acoustic descriptors are extracted and processed. A Cooperative Regression model provides an estimation of speaker’s perceived emotional status in terms of arousal and valence, and plots the detected emotions onto a circumplex diagram. *Lower part*: a schematic description of the functioning of the Cooperative Regression model. A new speech sample (unlabeled speaker) is fed to the algorithm for evaluation. Each Single Speaker Regression Model (SSRM) in the ensemble provides its own prediction about the new sequence. Dimensions and colors of the circles represent *heterogeneity* of the SSRM ensemble, which is a critical step into the model’s functioning and adaptability to different scenarios. Dimensions indicate that each SSRM can be trained on differently sized speech sequences and that descriptors extracted from each sequence can differ, as well as model parameters. Similarly, colors indicate that the emotional content can differ for each model. Predictions expressed by each SSRM are combined via a consensus rule. Newly labeled samples are added to the pool unless redundant (i.e. their content emulates that of samples already in the pool)(RED-EX criterion).

Here we describe a cooperative learning strategy (Fig 1) that simulates human decision-making in the social domain [20–22]. Group decision-making is a complex process that takes into account the distribution of individual preferences within a group and combines them to reach a collective response (e.g. Social Decision Scheme theory [23]). The combinatorial process can follow different decision schemes (e.g. majority vote, consensus, etc.) and may vary as a function of factors such as task and context. In the algorithm we propose, speech samples are fed to a classifier modeled to work as a group of individuals reaching a decision through a consensus scheme. In this analogy, consensus could be viewed as the means to extract the middlemost estimate or the ‘*vox populi*’ of Galton’s example. Specifically, for a set of speakers whose speech sequences and corresponding annotations were known, we trained and optimized separate regression models (Single Speaker Regression Model, SSRM). The ensemble of these models is shown in Fig 1 (left side), each colored circular block exemplifying one SSRM.

Unlabeled speakers (yellow question mark in Fig 1) are handled by the model as a human group would handle newcomers in a discussion: namely, individuals already part of the group make predictions on the opinions the newcomers may express. In the human case, different individuals exhibit different opinions, leading to a plethora of information. Similarly, in the algorithm each SSRM in the ensemble provides its own prediction about the unlabeled speech sequences. To combine these predictions we implemented a consensus rule: only responses exhibiting an average pair-wise concordance with the majority (*high consensus*) are retained and averaged to provide the final response (Machine Labeled Single Speaker Regression Model, ML-SSRM; situation C in Fig 1). We call this algorithm the Cooperative Regression Model (CRM) (Fig 1, central blue arrows).

In addition to select the SSRMs to be averaged, consensus is used here also to estimate reliability of the description assigned by the system to each unlabeled speech sequence. Inclusion criteria play a crucial role in the algorithm because new sequences—once labeled—are eventually added to the original pool. While this step ensures that the classifier’s capabilities increase with dimensions of the pool, it also requires a procedure that prevents insertion of unreliable sequences as well as that of sequences whose affective content emulates that of speakers already in the pool at the time of acquisition (*redundant speakers*). In fact, inclusion in the pool of a large number of closely similar sequences would produce (in the algorithm) a problem similar to that described for human groups: when conversations are dominated by a limited number of opinionated individuals, ‘group intelligence’ is largely reduced [24–26]. To minimize this risk, we introduced in the algorithm a redundancy exclusion criterion (REDundancy EXclusion, RED-EX; Fig 1 top right part) aimed at keeping the system free from self-referencing behavior. Specifically, in the model unlabeled speakers that meet the RED-EX criterion will be automatically excluded from the pool (redundant speaker, situation B in Fig 1). Finally, to avoid unproductive iterations we implemented selection of an optimal window width to calculate consensus level and added a dynamic consensus threshold for each window (i.e. the level of concordance sufficient to provide a reliable prediction is adaptively fixed based on intrinsic variation of the input data and related emotional content). That is, we simulated the groups’ choice of fixing a time limit for the debate and the fluctuations occurring in the members’ opinions as long as novel information becomes available.

In the following sections, we describe results from three tests performed on speech samples in order to compare performance of the present SER system to that of standard strategies. Results are discussed in terms of their theoretical and applicative implications. Indeed, it is easy to imagine one such system in laboratory settings (e.g. to match stimulus’ presentation to the speaker’s emotional state) or implemented in smartphones/tablets as an aid for patients unable to decode speakers’ emotional tone, or to facilitate activity of the numerous helplines.

## Materials and Methods

### Dataset

All simulations were performed on speech samples extracted from a public database (REmote COLlaborative and Affective interactions, RECOLA [27]). RECOLA is a speech corpus that was collected during a videoconference and contains spontaneous interactions between dyads of participants involved in a collaborative task [27]. The database includes 9.5h of speech recordings, collected continuously and synchronously from 46 participants. All collected speeches were in French, although participants differed as to their mother tongue (17 French, 3 Germans, 3 Italians). The total naturalness of speech recorded, makes RECOLA extremely suitable for our tests. As reported by RECOLA authors [27] twenty-three speakers gave informed consent to share their data and make their speech samples publicly available (10 male—13 female; mean age 21.3 ± 4.1 years). Only these samples were used in the present study. The non-consecutive numeric labels used to identify each speaker in the present work originate from the RECOLA dataset and are due to the fact that not all original speakers gave consent to use their multimodal emotional data. Emotional ratings for the RECOLA database were performed by six French-speaking assistants (3 females) for the first five minutes of all recorded sequences using a time-continuous annotation system for each affective dimension (ANNEMO web-based annotation tool, available at http://diuf.unifr.ch/diva/recola/annemo; for details see also Ringeval and co-workers [27]). As these were the only data made publicly available, these time periods were used here for training and testing.

### Metric

One of the crucial concepts in the developed strategy is concordance. Hence we firstly provide a quantitative description of a metric suitable to represent the concordance level between two sequences. In particular, given two time series *y*_1_(*t*) and *y*_2_(*t*), having mean values *μ*_y1_ and *μ*_y2_, and standard deviations *σ*_y1_ and *σ*_y2_, the Concordance Correlation Coefficient (CCC) [28] is computed as follows:

$$\text{CCC}=\frac{2\;\rho_{\text{y1y2}}\sigma_{\text{y1}}\sigma_{\text{y2}}}{[\sigma^{2}{}_{\text{y1}}+\sigma^{2}{}_{\text{y2}}+\left(\mu_{\text{y1}}-\mu_{\text{y2}}{)}^{2}\right]}$$ (1)

This metric accounts for biasing and signal correlation simultaneously so that it globally provides an estimation of temporal agreement. The CCC ranges between values of -1 (*perfect negative agreement*) and 1 (*perfect positive agreement*). A value of 0 corresponds to no agreement. The CCC will be used both in the consensus rule indicated in Fig 1 as well as in the system assessment.

### Procedure

All tests were carried out on a personal computer with an Intel Quad Core i7 processor using the Matlab environment. When working, the algorithm occupies less than 1% of CPU usage and carries out the classification procedure in less then 1sec. A schematic description of the method is shown in Fig 1. The new semi-supervised approach presented here is structured into three main blocks: i) construction of single SSRM using the labeled speech sequences, ii) implementation of a consensus strategy to derive machine-labels for each unlabeled sequence, and iii) enlargement of the pool with new models trained on machine labeled sequences. The consensus rule is in turn composed by three minor steps: ii-a) application of a cooperative aggregation rule for adaptively and dynamically averaging the responses provided by each SSRM, ii-b) construction of the Machine Labeled Single Speaker Regression Model (ML-SSRM), and ii-c) implementation of a REDundancy EXclusion (RED-EX) criterion to decide whether or not including the ML-SSRM in the pool.

According to this formulation, each speech sequence can be placed in one of three possible states: labeled, unlabeled, and waiting for inclusion. Labeled Speech sequences (LS) are those for which a reliable manual annotation is available. Unlabeled Speech sequences (US) are those that must be predicted, and for which machine-labels are estimated. Waiting-For-Inclusion (WFI) speech sequences are those for which machine-labels have been estimated and a model is trained but no decision about their inclusion has been taken. WFI sequences are subjected to the RED-EX criterion before a decision is reached. In the following sections, we detail each step of the method.

#### Step 1—Construction of single speaker regression model (SSRM)

Each single SSRM is trained and optimized on a labeled speech sequence *y*_L_(t) and the corresponding acoustic speech feature matrix *X*(t). Based on previous work [29], we consider the 65 acoustic low-level descriptors (LLD) and their first order derivatives (producing 130 LLD in total) that were used in the last two INTERSPEECH Computational Paralinguistic challengEs (ComParE 2013–2014) [30–31] using the open source extractor openSMILE (release 2.0) [32]. This feature-set includes a group of 4 energy related LLD, 55 spectral related LLD, and 6 voicing related LLD providing a description of speech in time-frequency as well as in voice quality domain (for more details on the ComParE feature set, refer to [33–34]).

Acoustic model learning of emotion requires estimation of a gold standard from time-continuous dimensions. Here, the gold standard is extracted from the available annotations using a concordance-based weighted average procedure that averages the six annotations after subtracting their weighted mean values. Weights used in the mean centering are pairwise average correlation coefficient of each annotation with the remaining ones. Optimization of each SSRM is performed by identifying the most significant features using a new quadrant-based procedure inspired by Russell’s circumplex model of affect [35–36], which describes emotions along a continuous two-dimensional domain in terms of valence and arousal. Based on what suggested for facial expressions [36–37], a dimensional model—which plots emotions along a continuous space—was preferred to a categorical one because it was more likely to accommodate the fluctuations of the human voice as well as its continuous presentation. Methodologically, the two-dimensional model applied here thus looked better fit to correlate acoustic features with valence and arousal using an independent procedure for each quadrant.

On this basis, features-selection (based on the Correlation Features Selection criterion) and gold standard synchronization (based on the estimation of the annotators’ reaction lag for the labelled speech sequences) are both implemented quadrant by quadrant. Features selected in segments of negative and positive valence (and similarly for arousal) are then concatenated so that a unique average reaction lag is computed for the whole output range and can be used for gold standard synchronization. A Partial Linear Regression model is trained using the resulting optimized setting, producing the corresponding SSRM for each labeled speech sequence. The procedure is repeated for each speaker in the initial pool. Mathematical details of the approach can be found in the S1 File.

#### Step 2—Construction of cooperative regression model (CRM)

**Fig 2:**
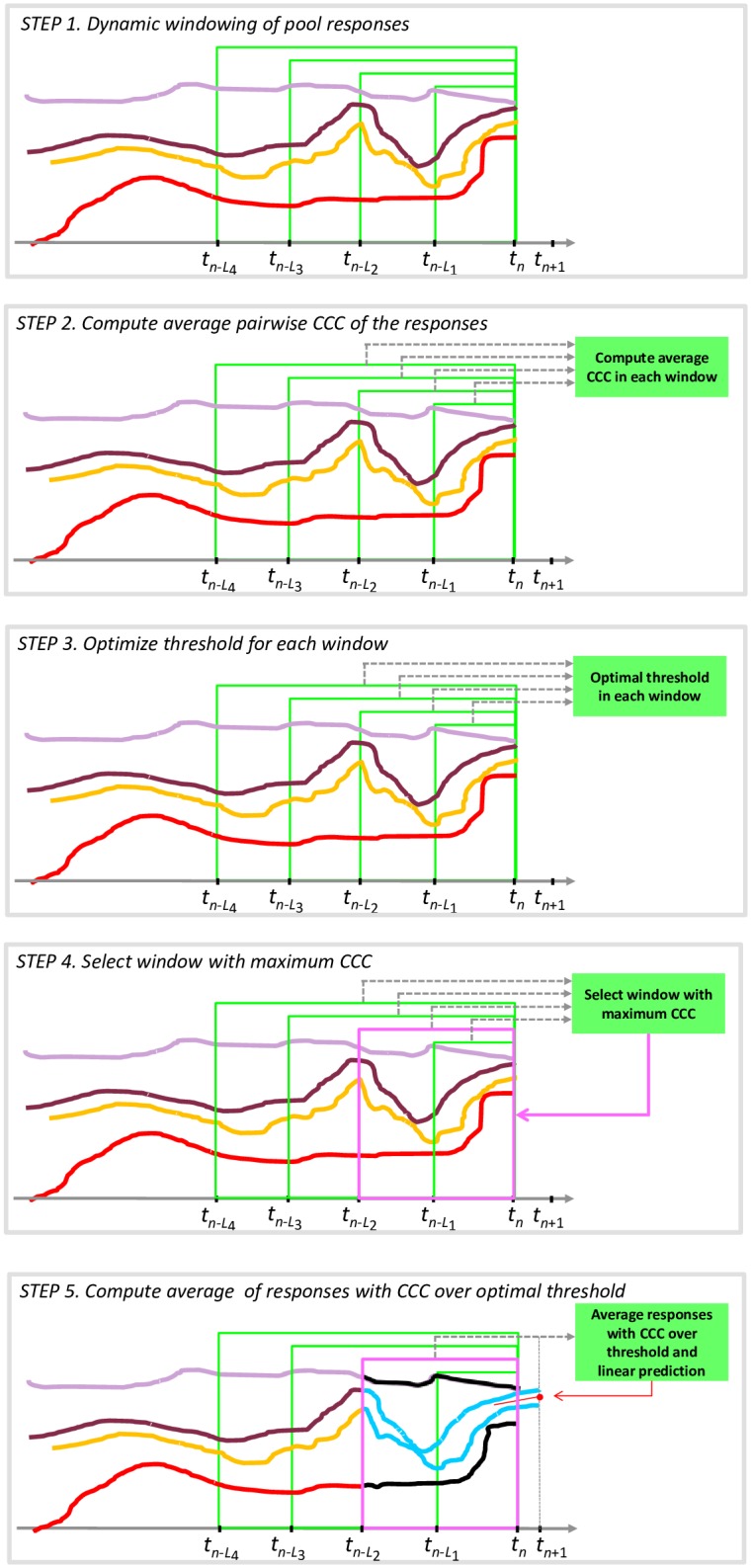
Graphical illustration of Steps 1–5. A schematic description of how the dynamic and adaptive cooperative strategy is implemented. Color legend as follows: for STEPS 1 to 4 the red, yellow, brown, and purple curves describe four synthetic predictions of a given output. For STEP 5, the cyan curves indicate the two concordant predictions. The black curves represent the less concordant prediction. Only the cyan curves are averaged to construct the unique final linear prediction (represented by the red segment superimposed on the rightmost part of the chart).

In order to test an unlabeled speech sequence (US), a cooperative aggregation rule is applied to the responses provided by each SSRM, performing steps 1–5 illustrated in Fig 2. In step 1 a dynamic windowing is applied to the available predictions, for a certain number of window widths: *L*_1_, …, *L*_N_. In step 2 the average pairwise concordance of each response with the others estimated in the same window is computed. In step 3, in order to select the most concordant responses among the available ones, a threshold is selected to maximize average concordance of the subset of responses with a CCC average over the threshold, minus the average concordance of the remaining subset of responses (i.e., this quantity can be seen as the sum of the average concordance of a group and the average disagreement of the remaining ones). Hence, step 3 produces the average CCC of concordant responses for each window. In step 4, the optimal window width is selected as the one presenting the maximum average CCC across all the tested window widths. Here, we considered window widths in the range [1s–8s] with a step size of 1s.

Finally, in step 5, provided the optimal window and the optimal threshold for that window, a linear fitting of the concordant responses over a time interval of 1*s* is used to extrapolate the predicted value at time *t*_n+1_ (identified by the red dot in the bottom panel of Fig 2). The procedure is repeated at each time instant of the entire observation period. If the optimal concordance is negative, the prediction is not computed and the output machine label at that time is missing.

Note that the model for the speaker under test is constructed only if the number of available predictions is sufficiently high; else the speaker is excluded from the pool (and set to the EXCLUDED state, Fig 1). In all other cases, the extracted machine labels are used to construct the corresponding SSRM following the procedure described in Step 1 and in the S1 File. We denote each of these models as Machine Labeled Single Speaker Regression Model (ML-SSRM). These models are set to the WFI state, meaning that they are reliable but not yet able to significantly improve system performance.

#### Step 3—Redundancy exclusion criterion (RED-EX)

In order to autonomously decide whether or not to add the ML-SSRM of the WFI sequence to the pool, the system evaluates the opportunity to increase the overall prediction capability of the system, while avoiding redundancy with respect to the models already in the pool. This is done by the REDundancy EXclusion (RED-EX) criterion, which compares the prediction of the model constructed on the WFI sequence with that provided by the SSRMs in the pool, using a further unlabeled speech sequence for testing. If the global concordance of the responses provided by the ML-SSRM with at least one response provided by the SSRMs in the pool is higher than 0.99 then the ML-SSRM does not add improvement to the pool. Therefore, the model is not included and set in the state EXCLUDED (Fig 1, situation B). Conversely, if the maximum concordance with the responses provided by the pool is lower than 0.99 then the new ML-SSRM is set to the state INCLUDED (Fig 1) and added to the pool. The procedure described in Step 2 is then applied to the enlarged ensemble to predict the affective content of the additional unlabeled speech sequence. The RED-EX criterion thus reduces the risk that speech samples that are too similar to those already present into the pool (i.e. samples which are unlikely to provide novel information) are included. In this way, biases in the labeling procedure that may result from poorly diversified information are prevented, and the computational burden is kept minimal.

#### Tests 1–3

In order to test the performance of the proposed strategy in a naturalistic speech environment and demonstrate its capabilities in larger scenarios, we performed three tests. In Test 1 performance of the present semi-supervised learning strategy was compared to that of a standard supervised approach using an identical initial set of labeled speech sequences. In Test 2 robustness of the algorithm was tested with respect to variations in entering order of the different speakers. In Test 3 sensitivity of the approach to self-referencing behavior was assessed by repeatedly providing the same speech sequence as input to the system.

To run these three simulations, we divided the RECOLA [27] dataset into two distinct sets, one for the construction of the SSRM of the initial pool and one for the testing of the semi-supervised regression strategy. In line with the assumption that in semi-supervised approach there is a small amount of labeled data and a relatively larger amount of unlabeled data, we randomly select a pool of four speakers from RECOLA. Using a “leave one speaker out” cross-validation strategy, we extracted the frequency of inclusion of each speaker in the training set for the prediction of the speaker in test. Based on the results, we selected speakers P23 (F), P30 (F), P43 (F), and P65 (M) that were averagely included (labels in parenthesis indicate gender). Moreover, based on preliminary simulation results, we eliminated speakers P16 (M), P17 (M), P34 (M), and P62 (M), whose features presented intrinsic problems that caused them to be rarely included in testing and to exhibit low prediction performance (due to a general emotional flatness in their speech). The remaining 15 speakers were used to validate the proposed semi-supervised strategy.

Performance was evaluated using the CCC metric defined above in order to quantify the discrepancy between expected and estimated responses for each speech sequence in test either in terms of signal correlation or in terms of mean square error. Boxplots of the CCC obtained over the 15 speakers in test are used to visualize the results, and when needed, t-test were performed to demonstrate the statistical significance of improvement achieved using the proposed strategy vs. the application of supervised learning strategy. Alpha level was set at .05 for all tests. For the sake of clarity, details for each of the three tests are provided in the corresponding Results sections.

## Results

### Test 1: Comparison of semi-supervised and supervised strategies

We compared the present model and a standard supervised approach by feeding them both with the same set of pre-labeled data. The supervised approach is assumed to systematically rely on the existing data pool. Accordingly, it is expected to be stably accurate but strongly dependent on the quality of labeled data. In other words, it mimics the behavior of a very conservative group of people, which—if prejudiced—may bias final results. Conversely, the semi-supervised method allows for on-line inclusion of any newly generated machine-labeled models, resembling more to a liberal group, open to novel opinions. Hence it should be more dynamic and explorative, looking for patterns that were not previously anticipated (but that might also result uninteresting).

As already mentioned, for the simulation, we used the annotated speech corpus RECOLA [27] and compared predictions on the emotional content of 15 test speakers as obtained by the two approaches. Acoustic emotional features were identified according to a quadrant-based procedure inspired by Russell’s circumplex model of affect [35–36] (see also S1 File), which represents emotions along a continuous two-dimensional domain in terms of valence and arousal. Performance of the two approaches was separately evaluated for arousal and valence in terms of Concordance Correlation Coefficient (CCC) [28], a type of metric that accounts for biasing and signal correlation simultaneously, providing an estimation of temporal agreement.

**Fig 3:**
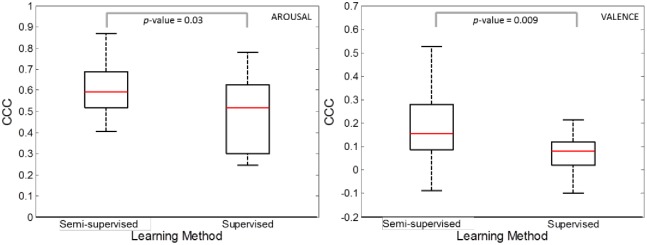
Box-plot of the concordant correlation coefficient (CCC) values for the semi-supervised strategy and supervised strategy. Results relating to arousal (left) and valence (right) were independently obtained.

The boxplot of CCC values obtained for arousal (left) and valence (right) are shown in Fig 3, together with *p*-values for the related t-tests. In the arousal dimension, the semi-supervised method strongly increased the CCC values (median ± interquartile range: 0.59±0.17) compared to the supervised system (0.52±0.33, t = 2.377, df = 14, p < .03), with variations up to 0.88. The same was true for the valence dimension, in spite of the lower values of CCC obtained (semi-supervised: 0.16±0.19; supervised: 0.08±0.10, t = 2.985, df = 14, p < .01). Namely, by allowing for on-line inclusion of additional information in the form of the newly generated machine-labeled models, the semi-supervised approach increased the sampling pool and significantly enhanced its predictive capabilities compared to the standard supervised model. A large set of accurately machine-labeled items, i.e. a ‘wise crowd’, led to a significantly improved performance.

### Test 2: Testing robustness to rearrangement of speakers in testing

Behavioral studies show that when competing options are presented in a sequence, order of appearance affects final evaluation. For example, when a jury evaluates a series of candidates, ratings increase with their serial position [38–39]. In the present model, order of testing could influence final performance due to the role played in the method by concordance (between responses provided by the pool) and redundancy (implemented by the RED-EX criterion, see also Test 3). To assess robustness of the model with respect to this issue, we quantified dispersion of the obtained CCC values when order of testing repeatedly changed in different iterated simulations (S1 Fig).

Ten different simulations were run by randomly rearranging order of speakers in testing and computing CCC values for each speech sequence in test when it appeared in a different position in the test sequence. Order of the sequences chosen at each iteration was the same for arousal and valence. As in Test 1, we compared performance under semi-supervised and supervised learning.

**Fig 4:**
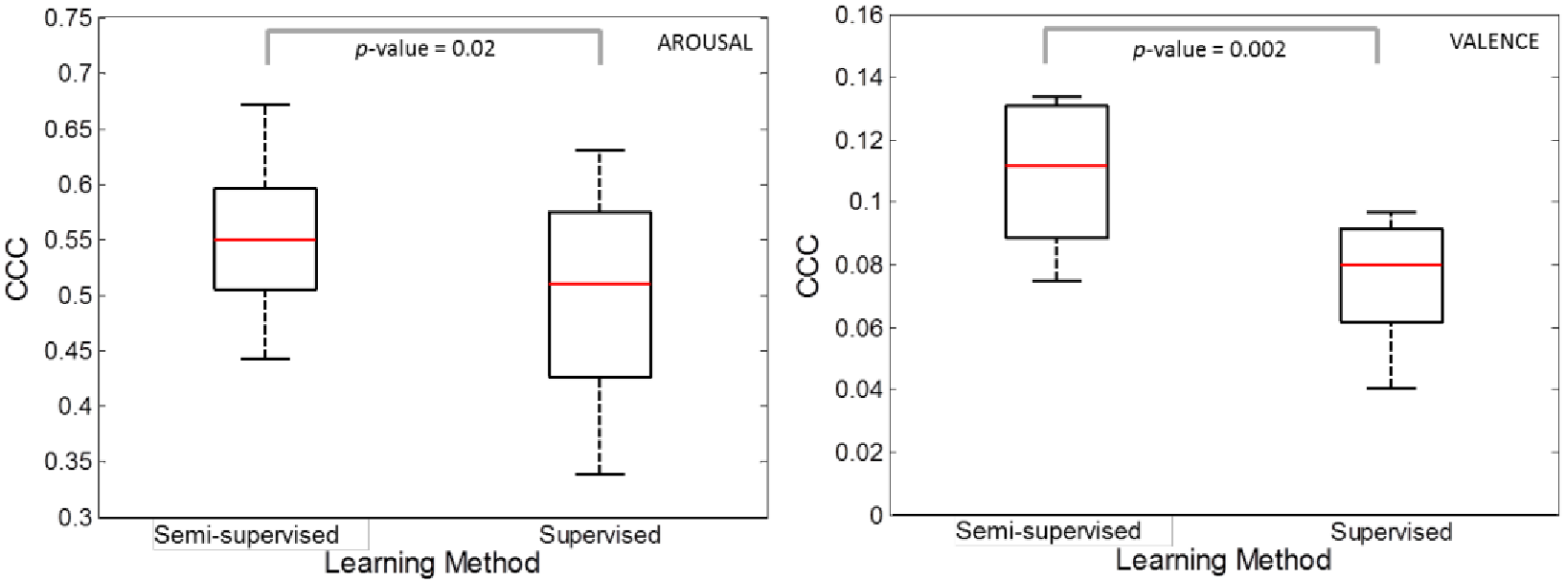
Effects of rearrangement of speakers’ order. Box-plot of the median concordant correlation coefficient (CCC) computed over the 15 speech sequences in test achieved during the 10 iterations for arousal (left) and valence (right) comparing semi-supervised and supervised strategies. P-values of the t-test (arousal: p < .02; valence: p < .002) demonstrate that the semi-supervised strategy significantly improves general performance of the emotion recognition system.

Fig 4 shows the boxplot of the median CCC values computed over the 15 speech sequences in test for the 10 iterations for arousal (left) and valence (right). A significant advantage was found for the semi-supervised compared to the supervised machine (arousal: t = 2.686, df = 14, p < .02; valence: t = 3.704, df = 14, p < .002). The effect was stronger for the valence compared to the arousal dimension. For both dimensions, predictions obtained under the semi-supervised method were less permeable to order effects and maintained better performances when compared to supervised strategy.

### Test 3: Testing robustness to inclusion of redundant speech sequences

Groupthink [24–25] is a phenomenon described in the social domain whereby failures in collective decision-making occur as a consequence of excessive cohesion within the group. This principle was nicely stated by social psychology in the 70s with reference to the Bay of Pigs invasion: the strong cohesion of the White House members either limited the possibility for alternative opinions or censored them, leading to a less objective (and fruitful) evaluation [24–25]. In the present model a groupthink analog can arise when an excessively narrow range of inputs is provided or when samples are too similar to one another (e.g. a speech sequence is inserted whose affective content emulates that of a speaker already in the pool). This could lead to self-referencing behavior and failure to generate correct labeling. To explore this issue, efficacy of the ad-hoc inclusion criterion, the RED-EX criterion, in preventing degeneration was tested as follows (S2 Fig).

**Fig 5:**
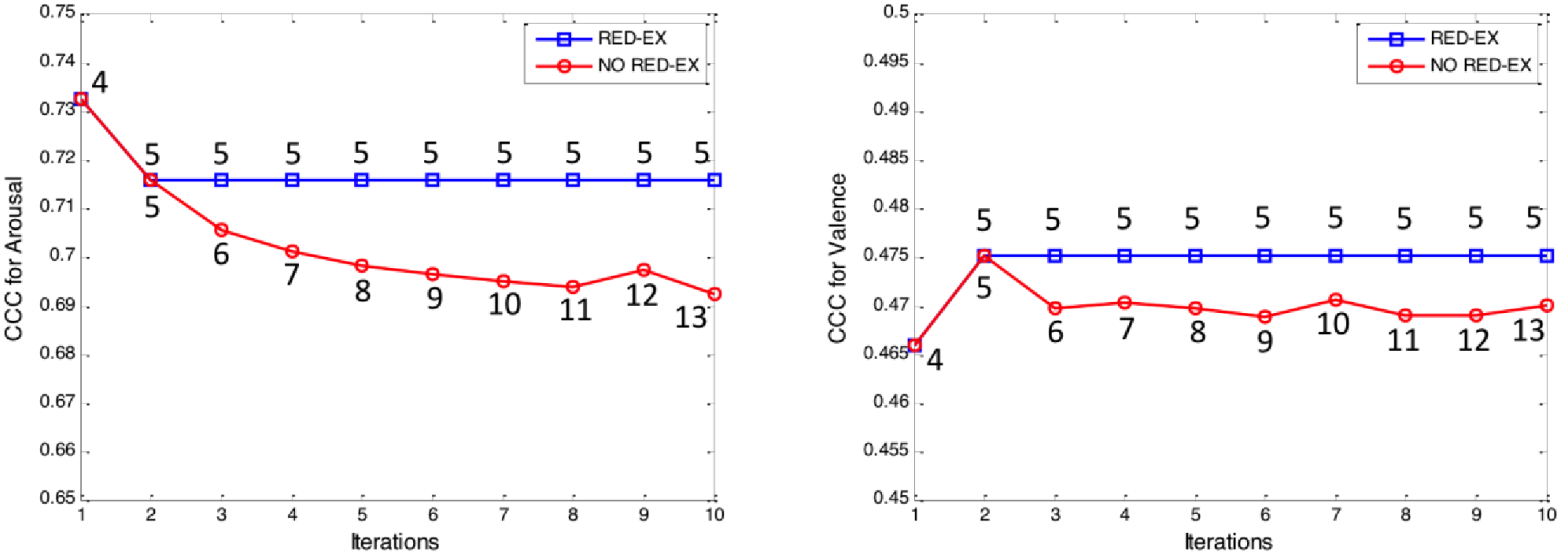
Effects of inclusion of redundant speech sequences. Comparison of CCC values for the prediction of arousal (left) and valence (right) according to whether the semi-supervised strategy was used in combination with the RED-EX criterion (blue lines) or not (red lines) in the Test 3. Numbers indicate cardinality of the pool at each iteration.

A series of simulations were run in which the system was repeatedly fed with a single speech sequence. The effects of enabling/disabling the RED-EX criterion were compared. Fig 5 reports CCC values of the prediction achieved for the speech sequence in repeated tests with respect to its gold standard (P20 in this case) for arousal (left) and valence (right), comparing the case when RED-EX criterion was applied (blue lines) or not applied (red lines). Numbers indicate cardinality of the pool at each iteration for the two conditions. Higher CCC values of predictions were obtained with the reduced pool (five models in both dimensions). Importantly, when the RED-EX criterion was not used, performance worsened during the repeated input of the same speaker (“*self-referencing*”). Conversely, when the RED-EX criterion was used, after an initial transient, performance remained stable around an optimal value, as desired. Indeed, a significant difference emerged for predictions relative to both arousal (t = 5.405, df = 9, p < .0004) and valence (t = 4.433, df = 9, p < .002) when the RED-EX criterion was used compared to when it was excluded. This confirms that the RED-EX criterion prevents the exponential increasing of models in the pool (which may occur during semi-supervised learning) by providing a guiding principle that selects which model to include for improving the system’s prediction capability.

## Discussion

We describe a novel speech emotion recognition system that applies a semi-supervised prediction model based on consensus. The system allows both for assigning new labels to unlabeled sequences and for preventing self-referencing. This approach deeply departs from procedures like active learning [10–40] (which aims at identifying an optimal pool of labeled sequences among the available ones) and from self-training [20] (that aims at enhancing robustness of existing classifiers, embedding labeled and unlabeled data). Novelty of the method lies in the modular architecture of the cooperative system, its strong reliance on the concept of concordance and the dynamic application of consensus rule. On these respects, the model calls forth the mechanisms involved in human group decision-making, of which shares the many benefits, including a substantial increase in accuracy as a function of sample size (the so-called *wisdom of the crowd* effect)[17–18,26]. As in a real crowd, the present algorithm is improved by the addition of novel inputs, particularly when they significantly differ from those already contained in the pool. This would be in line with observations on the detrimental effects that ‘groupthink’ [24–25] plays in humans, and with recent findings showing that ‘group intelligence’ is largely reduced when conversations are dominated by a limited number of individuals [26]. Differently from human groups (in which the same individuals produce *and* classify the emotional content), the algorithm described here mainly act as a classifier (although the newly added speech sequences, i.e. the signal generators, when added to the pool become, they too, classifiers).

Methodologically, the system largely differs from traditional approaches where the whole set of available data is sent as input to a prediction system (that embeds features extraction, reduction, and regression method). In these approaches, each addition to the training set is laborious, requiring re-training of the whole prediction system and, consequently, causing an exponential increase in both complexity and computational time. Conversely, the strategy proposed here is modular: accordingly, predictions over single speaker models are trained and labeled in parallel, benefiting from the advantages of cooperative learning. By simultaneously taking into account a plethora of opinions, the system increases its prediction capabilities and provides responses that go beyond mere averaging.

To account for parallel processing, the present method strongly relies on the concept of concordance. On this respect it differs from previously described SER systems, which mainly depend on correlation. The main advantage is that concordance shares with correlation the capability to assess the statistical discrepancy between random processes or time series, but additionally accounts for biasing. Biasing is a known distortion effect produced by systematic contributions: in the case of speech emotion recognition, it may lead to disastrous consequences when independently manifested in the two correlated dimensions of valence and arousal. Based on the Russell’s two-dimensional depiction of affect assumed here [35], different biasing terms, independently occurring in valence and arousal, will produce a displacement in the circumplex diagram that may completely alter the predicted emotional state. To prevent this issue, the present method relies on concordance correlation to assess for a realistic comparison between predicted and expected emotional dimensions, i.e. on a metric that simultaneously evaluates discrepancy between two signals in terms of both biasing and correlation. Concordance is used here also to prevent undesirable behaviors and implement the consensus rule. Specifically, we anticipated the need for a redundancy exclusion criterion to avoid self-referencing of the prediction system. This rule automatically prevents inclusion of machine-labeled models with no additional capability to predict future speech sequences than the pool itself. In addition, concordance is used for the first time in the implementation of a consensus rule. Predictions provided by each SSRM are collected and dynamically averaged according to their mutual consensus, extending the logic of majority voting to the regression framework. At each time instant, the most concordant predictions over a temporal delayed interval are averaged; in contrast, outlier predictions are excluded. In such a way, consensus among predictions is evaluated accounting for their mutual biasing and statistical correlations. Such a dynamic application of the consensus rule proposes a more realistic and complete logic of dynamic sliding windowing: namely, a model in which duration of the temporal window devoted to concordance evaluation changes dynamically according to the predictions provided by the individual regression models. Accordingly, a high level of concordance can be reached at once (due for example to obviousness of perceived emotions or capability of the models involved) or following a long listening session. As a matter of fact, dynamicity provides a benchmark for implementing *multimodal* emotion recognition because it allows for the possibility that different communicative channels may require different temporal intervals to express their informative content.

Central to our approach is the way semi-supervised strategy is implemented. During the past few years, semi-supervised learning appeared an unfeasible approach for a context as complex as that of emotion recognition. Difficulties in obtaining acceptable results with supervised strategies did not constitute, until now, a convincing starting point for experiencing semi-supervised strategies [41–42]. Hence the most recent attempts [20] investigated semi-supervised learning approaches mainly as a mean for optimizing performance and reducing the amount of human annotation via machine labeling. Our approach takes advantage from a restricted pool of labeled speech sequences to build a single regression model for each sequence in the pool. These models are now ready to predict in a dynamic cooperative way the emotional content of any new speech sequence. The final prediction, when meeting with a sufficiently high level of concordance, is deemed reliable to apply machine-labels to the novel (semi-supervised) sequences. Subsequently, the new models constructed on the machine-labeled sequences are fed to the initial pool. With this respect, the system is speaker-independent, as shown by the low dependency of the labeling processes from the order of testing used for presentation of new speech sequences to the pool.

To conclude, we present an entirely novel approach to the SER problem, which benefits from a slender and fast-processing architecture, and is enhanced rather than encumbered by increased sampling. At present, classification is provided only on a dimensional model, but we are confident that these data could be next used—if appropriately treated—to fit the framework of discrete emotion theories (such as Ekman’s)[43]. In addition, although the algorithm now exclusively targets speech, we expect that it could be eventually incorporated into a multimodal emotion prediction system, i.e. one that uses video, speech and physiological signals (such as ECG and/or EDA) that could extend and enrich prediction capabilities. For its characteristics, the system is likely to fit the demands of a number of situations, including use of portable voice analyzers, an advantage that could be of interest to cognitive science and rehabilitation. As to the former, application of this method to speech analysis approaches could be valuable in studies on patients for whom affective disturbances of speech are known, such as autism spectrum disorder [3–4] or Parkinson Disease [5–7]. In addition, fast processing qualifies this algorithm as a possible method for automatically pacing stimulus presentation and/or selecting data collection based on the speakers’ emotional tone, granting novel possibilities to neurophysiological and neuroimaging research. As to translational research, the opportunity to implement this SER system into a smartphone or tablet would provide a valuable aid to affective rehabilitation. Training sessions in which feedback is given by the system as to whether prosody and/or affective tone is adequate to a presented context would represent a useful tool for at-home therapy in the case of neurologic patients and an excellent alternative to the human-to-human interaction in autistic individuals. Finally, being devised as a simile to human behavior, this method could stimulate development of novel frameworks for simulating human behavior in contexts of consensus seeking, stereotypes construction and group decision-making (e.g. the “like” and viral effects in social networks and similar phenomena).

## Supporting Information

S1 Fig Schematic illustration of Test 2 to test the robustness to rearrangement of the order of speech sequences in testing. The same set of three sequences (on the left) are repeatedly rearranged and input to the semi-supervised learning machine based on the cooperative regression and RED-EX criterion. The performance in terms of CCC values for the comparative simulations are reported in the main text.
(DOCX)

S2 Fig Schematic representation of the test run to demonstrate the effectiveness of the RED-EX criterion to prevent self-referencing. The cooperative regression system is fed with an unlabeled speech sequence (green circle on the left) that is already in the pool of labeled speakers (feed 1). The cooperative regression module applies on it, generate the M-labeled speech sequence, but then put it in the WFI condition (gray circle on the right). At this point the RED-EX criterion applies and evaluates if the inclusion of the new annotated speech sequence may add improvement to the system knowledge-base by computing its CCC with the speech sequences already in the pool. A too high CCC value makes the RED-EX criterion been verified and the sequence excluded (red arrow in the bottom-left). The feed is repeated (feed 2) with the same input speech sequence but without the application of the RED-EX criterion. In such case, after been machine-labeled the sequence is included in the cooperative model. To emphasize the effect, the two kind of simulation are repeated for 10 times each.
(DOCX)

S1 File Supplementary Methods. In this Section, we provide mathematical details of the main steps involved in the construction of each Single Speaker Regression Model (SSRM).
(DOCX)
