# Supplementary material for: Strength Is in Numbers: Can Concordant Artificial Listeners Improve Prediction of Emotion from Speech?
Source: PLoS One. 2016 Aug 26;11(8):e0161752. doi: 10.1371/journal.pone.0161752 (PMC5001724; doi:10.1371/journal.pone.0161752)
Supplement: S1 Fig — The same set of three sequences (on the left) are repeatedly rearranged and input to the semi-supervised learning machine based on the cooperative regression and RED-EX criterion. The performance in terms of CCC values for the comparative simulations are reported in the main text. (DOCX) [file pone.0161752.s001.docx]

Strength Is In Numbers: Can Concordant Artificial Listeners Improve Prediction Of Emotion From Speech?

Eugenio Martinelli^1^, Arianna Mencattini^1^, Elena Daprati^2,3*^ & Corrado Di Natale^1^

Supporting Information – Fig. S1

**Graphical representation of the implementation of test 2**

**Figure S1** provides a graphical representation of simulations performed in test 2 to demonstrate the robustness of the method to changes in order of testing of the unlabeled speech sequences.

**Figure S1: Schematic illustration of Test 2 to test the robustness to rearrangement of the order of speech sequences in testing.**

The same set of three sequences (on the left) are repeatedly rearranged and input to the semi-supervised learning machine based on the cooperative regression and RED-EX criterion. The performance in terms of CCC values for the comparative simulations are reported in the main text.
