# Supplementary material for: Strength Is in Numbers: Can Concordant Artificial Listeners Improve Prediction of Emotion from Speech?
Source: PLoS One. 2016 Aug 26;11(8):e0161752. doi: 10.1371/journal.pone.0161752 (PMC5001724; doi:10.1371/journal.pone.0161752)
Supplement: S2 Fig — The cooperative regression system is fed with an unlabeled speech sequence (green circle on the left) that is already in the pool of labeled speakers (feed 1). The cooperative regression module applies on it, generate the M-labeled speech sequence, but then put it in the WFI condition (gray circle on the right). At this point the RED-EX criterion applies and evaluates if the inclusion of the new annotated speech sequence may add improvement to the system knowledge-base by computing its CCC with the speech sequences already in the pool. A too high CCC value makes the RED-EX criterion been verified and the sequence excluded (red arrow in the bottom-left). The feed is repeated (feed 2) with the same input speech sequence but without the application of the RED-EX criterion. In such case, after been machine-labeled the sequence is included in the cooperative model. To emphasize the effect, the two kind of simulation are repeated for 10 times each. (DOCX) [file pone.0161752.s002.docx]

Strength Is In Numbers: Can Concordant Artificial Listeners Improve Prediction Of Emotion From Speech?

Eugenio Martinelli^1^, Arianna Mencattini^1^, Elena Daprati^2,3*^ & Corrado Di Natale^1^

Supporting Information – Fig. S2

**Graphical representation of the implementation of test 3**

**Figure S2** provides a graphical representation of simulations performed in test 3 to demonstrate the robustness to redundant speech sequence inclusion.

**Figure S2: Schematic representation of the test run to demonstrate the effectiveness of the RED-EX criterion to prevent self-referencing.**

The cooperative regression system is fed with an unlabeled speech sequence (green circle on the left) that is already in the pool of labeled speakers (feed 1). The cooperative regression module applies on it, generate the M-labeled speech sequence, but then put it in the WFI condition (gray circle on the right). At this point the RED-EX criterion applies and evaluates if the inclusion of the new annotated speech sequence may add improvement to the system knowledge-base by computing its CCC with the speech sequences already in the pool. A too high CCC value makes the RED-EX criterion been verified and the sequence excluded (red arrow in the bottom-left). The feed is repeated (feed 2) with the same input speech sequence but without the application of the RED-EX criterion. In such case, after been machine-labeled the sequence is included in the cooperative model. To emphasize the effect, the two kind of simulation are repeated for 10 times each.
