# Supplementary material for: Strength Is in Numbers: Can Concordant Artificial Listeners Improve Prediction of Emotion from Speech?
Source: PLoS One. 2016 Aug 26;11(8):e0161752. doi: 10.1371/journal.pone.0161752 (PMC5001724; doi:10.1371/journal.pone.0161752)
Supplement: S1 File — In this Section, we provide mathematical details of the main steps involved in the construction of each Single Speaker Regression Model (SSRM). (DOCX) [file pone.0161752.s003.docx]

Strength Is In Numbers: Can Concordant Artificial Listeners Improve Prediction Of Emotion From Speech?

Eugenio Martinelli^1^, Arianna Mencattini^1^, Elena Daprati^2,3*^ & Corrado Di Natale^1^

Supporting Information – file S1

**Supplementary Methods**

**Construction of the Single Speaker Regression Model (SSRM)**

In this Section, we provide mathematical details of the main steps involved in the construction of each Single Speaker Regression Model (SSRM). The description will be provided for a generic affective dimension (arousal or valence) for simplifying the notation.

**Gold Standard Estimation**

For each speech sequence professional raters provided six different annotations, $y_{r_{i}}$ $i=1,\ldots,N_{r}$ being $N_{r}$ the number of raters (six in this experiment). In order to extract a unified gold standard annotation, we performed a weighted average of the six ratings, using concordance as guiding principle. Formally, the six evaluations are shifted of the same quantity $\bar{y}$ as obtained by applying Eqs. (1) - (3)

$\bar{\rho}\left( i \right)=\frac{1}{N_{r}}\sum_{j=1,j\neq i,\rho\left( i,j \right)>0}^{N_{r}} \tilde{\rho}\left( i,j \right)$ (1)

$\bar{y}=\frac{1}{\sum_{i=1}^{N_{r}} \bar{\rho}\left( i \right)}\sum_{i=1}^{N_{r}} \frac{1}{T}\sum_{t} y_{r_{i}}(t)\bar{\rho}\left( i \right)$ (2)

$y\left( t \right)=\frac{1}{N_{r}}\sum_{i=1}^{N_{r}} \left( y_{r_{i}}\left( t \right)-\bar{y} \right)$ (3)

with $\bar{\rho}(i)$ indicating the mean pairwise Pearson’s correlation coefficient of the annotation provided by the evaluator $r_{i}$ with the remaining $N_{r}-1$, and $\tilde{\rho}\left( i,j \right)=max\left( 0, \rho\left( i,j \right) \right)$being the positive Pearson’s correlation coefficient between ratings $r_{i}$ and $r_{j}$*.* Hence, in averaging annotations, such procedure gives priority to the raters that agree with the pool.

**Feature selection**

The two fundamental guiding principles of feature selection occurring during the construction of each SSRM are input-output synchronization and correlation based selection. It has been demonstrated that raters need some time to evaluate the cues observable in an audiovisual sequence and then report the corresponding emotion, especially on time-continuous ratings of emotion. The estimation of the so-called reaction lag (RL) depends on the speaker, on the affective content under observation, and on the rater of course. In order to simplify the procedure of estimating the RL, and hence synchronizing features and annotation according to this, we assumed as negligible the variation of the RL among the six raters of the same speech sequence. In order to reduce the variation of the emotional content of speech sequences during synchronization, we we preliminary segmented the features and corresponding annotations according to the quadrant they belong in the circumplex Russel diagram of affect, i.e., positive or negative arousal, positive or negative valence. The procedure is called Quadrant Based Temporal division (QBTD). In detail, denoting with *q*  the generic affective dimension (arousal or valence), we denoted with $y_{q+}\left( t \right)$and $y_{q-}\left( t \right)$ the gold standard segments of positive and negative dimension and with $x_{q+}^{k}\left( t \right)$ and $x_{q-}^{k}\left( t \right)$ the corresponding segments for each feature $x^{k}$. Denote now with $C_{x^{k}x^{j}}$ the correlation matrix of feature vectors and with $C_{x^{k}y}$ the cross-correlation matrix of each feature $x^{k}$ with the annotation *y*. The feature selection procedure is divided into two steps:

- Sort the element in the correlation matrices according to descending order, obtaining $\tilde{C}_{x^{k}x^{j}}$ $\tilde{C}_{x^{k}y}$

- Take the first K sorted features that maximize the correlation feature selection (CFS) quantity

$CFS=\frac{\sum_{k=1}^{K} \tilde{C}_{x^{k}y}}{\sqrt{K+2\sum_{k=1}^{K} \sum_{j=k+1}^{K} \tilde{C}_{x^{k}x^{j}}}}$ (4)

The following steps are applied for the synchronization and feature selection procedure:

i) shift each gold standard segment *y* of a lag RL in the range [0*,* 8] *s*, with a step size of 40*ms*;

ii) for each lag compute the *CFS* using Eq. (4);

iii) choose the optimal lag that produces the maximum *CFS*;

iv) choose the features related to the optimal lag.

Note that, the procedure described in steps: i)-iv) for the selection of the optimal RL values are repeated for each quadrant and for each affective dimension. At the end, the RLs obtained for the two quadrant of the same affective dimension are averaged and the synchronization is performed using the average RL achieved. In a similar way, the features selected in different quadrants of the same affective dimension are unified in the same set of features selected.

**Linear Regression**

Each SSRM is finally built by training partial least square regression (PLS) on the selected synchronized features and gold standard annotations. First the features selected are normalized by auto-scaling with normalization parameters, mean value $\mu_{x^{k}}$ and standard deviation $\sigma_{x^{k}}$. The optimal number of latent variables *LV* in the PLs model for valence and arousal are extracted through contiguous block splitting cross-validation performed on the entire speech of the speaker under training, with 50 splits (each of an approximate length of 6 s). The SIMPLS algorithm is used to estimate the PLS coefficients *b*_n_, n=1,…,*N*_x_ being *N*_x_ the number of features selected for a speaker in a given affective dimension. The final predicted value for the generic output, $\hat{y}\left( t \right)$*,* is obtained as

$\hat{y}\left( t \right)=X\left( t \right)\cdot B+B_{0}$ (5)

where the vector $X(t)$ contains , at each instant *t*, the features vector selected for that affective dimension and $B=[b_{1},b_{2}, \ldots b_{n}]$. The terms $B_{0}$ are used for rescaling the output response after mean centering.
